# Impact of I-PASS handover training on compliance and communication quality: a clinical audit at a teaching hospital

**DOI:** 10.1097/MS9.0000000000005694

**Published:** 2026-09-21

**Authors:** Malaz Kamaleldin Omer, Yousuf Alnoor Younis Mohammed, Hibatalla Elamin Hussein Adam, Mazin Abdalaal Hussien Helali, Mohamed Elsheikh Farah Mohamed Elhassan, Mahmoud Osman Mohammed Suleiman, Sirageldeen Abdallah Hamza Khidir, Morouj Hussien Mohammed, Ibtehal Abdalwahid Abdalmjeed Omer, Husam Aldeen Atif Abdolraheem Mohammed, Osman Mohammed Osman Abdallah, Eilaf Fadel Elmawla, Mohammed Osman Ahmed Osman, Gasm Alseed Abdelmonim Gasm Alseed Fadlalmoula

**Affiliations:** a Department of Internal Medicine, Sudan Medical Specialization Board, Khartoum, Sudan; b Department of Internal Medicine, Imperial University College, Khartoum, Sudan; c Department of Internal Medicine, University of Gadarif, Gadarif, Sudan; d Department of Internal Medicine, National Ribat University, Khartoum, Sudan; e Department of Internal Medicine, Alzaiem Alazhri University, Khartoum, Sudan; f Department of Internal Medicine, Alfashir University, North Darfur, Sudan; g Latifa Women and Children Hospital, Ministry of Health, Dubai, United Arab Emirates; h Department of Internal Medicine, Elmik Nimir Hospital, Shendi, River Nile State, Sudan

**Keywords:** clinical audit, clinical handover, emergency department, I-PASS framework, patient safety

## Abstract

**Background:**

Clinical handover between the emergency department (ED) and inpatient units is a high-risk process prone to communication errors and patient harm. This audit evaluated ED-to-internal medicine handover practices and assessed the impact of implementing an I-PASS-based standardized framework.

**Methods:**

A prospective closed-loop clinical audit was conducted at a public teaching hospital in Sudan in April 2024. Baseline data were collected via a structured questionnaire assessing compliance with I-PASS criteria, with a target standard of 100%. Thirteen questionnaire responses were analyzed in the first cycle. Following gap identification, a structured bedside I-PASS handover was implemented and reassessed in a second cycle.

**Results:**

Baseline handovers were informal and unstructured, with no standardized tool in use. All handovers were verbal; 30.8% occurred via telephone. Critical safety elements were inconsistently communicated: pending investigations were mentioned in 15.4% of cases, isolation precautions in 7.7%, risk identification in 46.2%, and next-step review in 38.5%. Patient or family involvement occurred in only 7.7% of cases. Following I-PASS implementation, structured bedside handovers were universally adopted. Core clinical elements achieved complete or near-complete compliance. Patient or family involvement increased from 7.7 to 92.3%.

**Conclusion:**

Baseline handover practices were variable and nonstandardized. I-PASS implementation was associated with improved handover compliance and perceived communication quality across all measured domains. Standardized handover is feasible in resource-limited settings, although further studies incorporating clinical outcome measures are needed.

## Introduction

Medical errors continue to be a major global concern, drawing the attention of healthcare professionals, policymakers, and the general public^[^1^]^, as they are a leading cause of morbidity and mortality. According to the Institute of Medicine (IOM) report To Err Is Human, adverse events affected 2.9–3.7% of 33 million people, with an estimated 44 000–98 000 hospital deaths annually attributable to preventable adverse events, ranking medical errors as the eighth leading cause of death in the United States, surpassing breast cancer, acquired immunodeficiency syndrome, and motor vehicle accidents^[^2^]^. Despite efforts to improve patient care since the report, medical errors continue to occur within the medical community. Emergency departments (EDs) are especially vulnerable to adverse events due to a combination of factors such as high patient acuity, frequent crowding, numerous interruptions, limited continuity of care, and increasing patient volumes. These challenges create an environment in which communication breakdowns are more likely to occur and can have serious consequences^[^3,4^]^. Addressing these issues requires a multifaceted approach that includes improved staff communication training, better resource allocation, and the use of technology to support decision-making. Recognizing the complexities of the ED environment enables healthcare providers to develop targeted strategies to reduce medical errors and improve overall patient outcomes.

HIGHLIGHTS

- Baseline handover compliance was critically low, with no structured format in use.
- I-PASS framework achieved 100% protocol-based bedside handovers.
- All five I-PASS domains reached near-complete compliance in Cycle 2.
- Patient and family involvement increased from 7.7 to 92.3% post-intervention.
- A low-cost intervention proved effective in a resource-limited hospital.

Clinical handovers are a critical point of vulnerability in the patient care pathway. The Australian Medical Association (AMA) defines handover as the transition of care that involves the exchange of information, responsibility, and authority between healthcare professionals^[^5^]^. Inadequate handover has been linked to poor clinical outcomes, such as inaccurate assessment and diagnosis, duplication or delay of investigations, medication errors, longer hospital stays, in-hospital complications, and lower patient satisfaction^[^6^]^. The risks associated with ineffective handover are amplified in the ED setting, where transitions occur frequently and often under significant time pressure. In 2006, in response to mounting evidence of harm caused by poor communication, the Joint Commission established standardized handover as a National Patient Safety Goal^[^7^]^. Similarly, the Royal College of Physicians has emphasized the need for structured handover in acute care settings^[^8^]^. Additional strategies for safe handover include using electronic medical records to provide structured data and reduce reliance on memory^[^9^]^, as well as actively involving receiving clinicians in the sign-out process to promote clarification and shared understanding^[^10^]^. In ED settings, handover occurs in various contexts, ranging from patient presentation via emergency transport, through shift changes within the ED, to admission to inpatient care. This clinical audit focuses specifically on handover practices between the ED and the internal medicine inpatient unit.

The aim of this audit is to improve the transition from the ED to the medicine inpatient unit at the study institution. The goals are to evaluate current handover practices used by ED physicians and internal medicine residents, identify strengths and areas for improvement, and implement evidence-based strategies to enhance the safety and reliability of information transfer, thereby indirectly contributing to improved patient safety and outcomes.

## Methodology

### Study design

This study was a prospective closed-loop clinical audit that aimed to evaluate and improve the quality of handover practices between the ED and the internal medicine inpatient unit.

### Study setting

The audit was carried out at a public teaching hospital in Sudan in April 2024.

### Study population

The target population included all physicians involved in the handover process between the ED and the Internal Medicine Unit during the audit period. This encompassed attending physicians (senior specialists), registrars (specialty trainees), medical officers (junior doctors), and house officers (interns) working in both departments. While nurses play a crucial role in the overall patient care transition, this specific audit focused on physician-to-physician handover practices.

### Sample size

The study aimed for total coverage of all eligible doctors participating in the handover process during the study period. A minimum target of 30 completed questionnaires was set to ensure a representative sample of physician perspectives. Prior to formal data collection, the questionnaire was pilot-tested on a small group using 10 handover encounters to assess item clarity and validity, but these were not included in the final analysis. The final version of the questionnaire was reviewed by three expert consultants with experience in clinical research to confirm face validity and cultural suitability; it was deemed to identify key patterns and areas for immediate intervention, aligning with methodologies used in similar exploratory observational studies^[^11^]^.

### Audit criteria and standards

Audit Criteria and Standards – Audit criteria were developed using the I-PASS framework and international handover guidelines, such as those from the Australian Commission on Safety and Quality in Health Care (ACSQHC), the Joint Commission International (JCI), and the World Health Organization (WHO)^[^12–14^]^. The assessment centered on the five core domains. Given the importance of handover communication and its direct impact on patient safety, the goal for each criterion was set at 100% compliance (Table 1). While acknowledging the complexities of the educational environment, this high standard provides a clear target for quality improvement efforts.

**Table 1:** Audit criteria and standard.

Criteria	Standard	Exception	Guidelines
Illness severity	100%		ACSQHC
Patient summary	100%		ACSQHC
Action list	100%		ACSQHC
Situational awareness and contingency plan	100%		ACSQHC
Synthesis by receiver	100%		ACSQHC

## Established guidelines and the I-PASS framework for effective handovers

Effective clinical handovers, or the transfer of patient care responsibility between healthcare providers, are critical milestones in a patient’s treatment journey. Unfortunately, communication breakdowns during these handovers can result in misunderstandings, errors, and negative outcomes^[^12^]^. The good news is that healthcare organizations can significantly improve patient outcomes by implementing standardized handovers that follow established best practices and structured frameworks.

### Established guidelines

National and international organizations provide a strong foundation for effective handovers:

1. **National Safety and Quality Health Service Standards (ACSQHC)**^[^12^]^: These standards emphasize dedicated handover times, clear communication tools, and patient and family inclusion. This aligns with I-PASS's focus on information sharing during a dedicated handover period.
2. **JCI**^[^13^]^: JCI promotes teamwork and communication, fostering a collaborative handover environment. While it does not offer specific handover standards, its focus on these areas is valuable. This collaborative approach aligns with the I-PASS emphasis on active listening and clear communication between providers during handovers.
3. **WHO**^[^14^]^: The WHO prioritizes medication safety, offering valuable resources that can be indirectly applied to improve handover content, especially for high-risk medications. This focus on clear and accurate medication information aligns with the I-PASS recommendation for a dedicated Action List outlining medication administration needs.

### The I-PASS framework for structured communication

The I-PASS framework provides a structured approach to conducting clinical handovers, focusing on key information exchange to promote clear communication and reduce the risk of misunderstandings. I-PASS stands for the following.

I. Illness Severity

P: Patient Summary

A: Action List

S: Situational Awareness and Contingency Planning

S: Synthesis by the Receiver

## Supporting I-PASS with established principles

The I-PASS framework is supported by a substantial body of evidence. Starmer et al. found that using the I-PASS mnemonic standardized verbal handoffs and improved communication clarity among clinicians^[^15^]^. A landmark multicenter study found that the implementation of an I-PASS-based resident handoff bundle significantly reduced medical errors and preventable adverse events among hospitalized children^[^16^]^. Building on these findings, the framework has been disseminated and adapted in a variety of clinical settings, with evidence demonstrating its positive impact on communication quality, interdisciplinary collaboration, and patient safety outcomes^[^17^]^.

## Incorporating I-PASS

With guidelines and a structured framework like I-PASS, healthcare organizations can significantly improve the effectiveness of clinical handovers, leading to better patient outcomes. The I-PASS Patient Safety Institute provides resources and training programs to support the implementation of this framework: https://www.ahrq.gov/teamstepps-program/curriculum/communication/tools/ipass.html.

## Data collection

### Data were collected in two phases

#### First cycle (baseline assessment)

Baseline data were gathered using a structured, self-administered questionnaire distributed electronically via Google Forms. The first cycle yielded thirteen completed responses. Data collected included the timing and location of the handover. Participants included, method of communication (verbal, written, or both), use of structured communication tools, and communication of key clinical elements. Inclusion of patients and families, and quality of communication (clarity, brevity, thoroughness) were rated on a 5-point Likert scale.

### Intervention

After analyzing the baseline data, a structured I-PASS-based handover intervention was implemented. A senior departmental consultant delivered training in two face-to-face workshops lasting about 1–2 hours each. The sessions were attended by all 13 participants who had completed the baseline questionnaire, as well as other colleagues. The intervention involved standardization of bedside handovers with emphasis on structured communication using I-PASS components; reinforcement of receiver synthesis. Promoting multidisciplinary participation encouraged patient and family involvement. The training covered the rationale for standardized handover as well as the five I-PASS components (illness severity, patient summary, action list, situation awareness and contingency planning, and synthesis by receiver), and participants received a printed I-PASS checklist to use at the bedside. The second audit cycle took place approximately 1 week after the training sessions.

### Second cycle (re-audit)

A second data collection cycle was carried out following the implementation to reassess compliance with the predefined standards using the same tools and criteria. Despite a target of 30 questionnaires per cycle, only 13 were returned (response rate: 43.3%), owing primarily to staff availability constraints, shift patterns, and competing clinical workloads. Notably, the same 13 participants responded in both cycles, resulting in a paired longitudinal design that improves the validity of the before-and-after comparison by accounting for inter-individual variability.

## Data analysis

The data were analyzed descriptively using IBM SPSS Statistics (version 26). Frequencies and percentages were calculated for each handover domain during both audit cycles. A descriptive comparison of cycles 1 and 2 was performed to identify trends in compliance improvement.

## Results

A total of thirteen responses were examined. The first cycle saw 69.2% compliance with standard handover time, with 0.0% occurring after duty. Bedside handovers occurred in 69.2% of cases, with phone handovers occurring in 30.8%. All handovers were carried out verbally, with no use of combined verbal and written methods or structured tools such as I-PASS (0%). Patient and family involvement was reported in 7.7% of cases.

The second audit cycle showed a significant improvement in the handover process over the first cycle. The second cycle saw 100% compliance with the standard handover time, with all handovers taking place after duty. Bedside handovers increased to 100%, while phone-based handovers decreased to 0%. Verbal-only handovers decreased to 0%, while structured tools (e.g., I-PASS) were used in 100% of cases, with no reports of combined verbal and written methods. Patient and family involvement rose to 92.3%, as shown in Table 2.

**Table 2:** Comparison of handover practices between first and second audit cycles.

Items	1st cycle (%)	2nd cycle (%)
Standard time for handover	69.2	100.0
Handover time (if standard): post duty	0.0	100.0
Handover location: patients’ rooms (bedside)	69.2	100.0
Handover location: through the phone	30.8	0.0
Handover method: verbally only	100.0	0.0
Handover method: both (verbal and written)	0.0	0.0
Common structure/rules used (e.g., I-PASS)	0.0	100.0
Patient and family included	7.7	92.3

In the first cycle, participation in handover was highest among registrars and medical officers (38.5% each), followed by house officers (23.1%) and attending physicians (15.4%). In the second cycle, all staff categories (attending physicians, registrars, medical officers, and house officers) participated in 100.0% of handovers, as shown in Table 3.

**Table 3:** Handover participants (ED department).

Items	1st cycle (%)	2nd cycle (%)
Attending physician	15.4	100.0
Registrars	38.5	100.0
Medical officer	38.5	100.0
House officers	23.1	100.0

In the first cycle, participation in handover was highest among registrars (69.2%), followed by house officers (23.1%) and attending physicians (15.4%), while medical officers had the lowest participation (7.7%). In the second cycle, all staff categories (attending physicians, registrars, medical officers, and house officers) participated in 100.0% of handovers, as shown in Table 4.

**Table 4:** Handover participants (medicine department).

Items	1st cycle (%)	2nd cycle (%)
Attending physician	15.4	100.0
Registrars	69.2	100.0
Medical officer	7.7	100.0
House officers	23.1	100.0

In the first cycle, illness severity and patient demographics (age, gender, and relevant PMH/PSH) were communicated in 100.0% of cases. Other elements showed variable reporting, including differential diagnosis (84.6%), chief complaint and timeframe (69.2%), review of systems and physical findings (61.5%), diagnostic results (61.5%), medications administered (61.5%), consults made (38.5%), suggested plan (53.8%), pending tasks (15.4%), potential risks (46.2%), isolation precautions (7.7%), receiving doctor’s summary (53.8%), and review of next steps (38.5%). In the second cycle, all elements were reported in 100.0% of cases except consults made (84.6%) and both potential risks and isolation precautions (92.3%), as shown in Table 5.

**Table 5:** Comparison of clinical handover content elements between first and second audit cycles (%).

Element	1st cycle (%)	2nd cycle (%)
Illness severity communicated	100.0	100.0
Age, gender, relevant PMH/PSH mentioned	100.0	100.0
Differential diagnosis provided	84.6	100.0
Chief complaints and timeframe communicated	69.2	100.0
Relevant review of symptoms and physical exam findings included	61.5	100.0
Diagnostic results communicated	61.5	100.0
Medications administered (incl. IV access) listed	61.5	100.0
Consults made mentioned	38.5	84.6
Suggested plan communicated	53.8	100.0
Pending studies, treatments, exams, procedures communicated	15.4	100.0
Potential risks and complications identified	46.2	92.3
Need for isolation precautions addressed	7.7	92.3
Receiving doctor summarized most likely diagnosis	53.8	100.0
Next steps/action list reviewed by receiver	38.5	100.0

In the first cycle, handover quality showed moderate performance, with clarity most frequently rated at level 3 (54%), brevity distributed across levels 1–4, and thoroughness predominantly at level 3 (46%). In the second cycle, ratings improved noticeably, with clarity, brevity, and thoroughness mostly rated at level 4, and smaller proportions at level 3, indicating more consistent and higher-quality handovers. Overall, the data demonstrate a clear improvement in handover performance across all domains, with a shift from moderate to higher ratings in clarity, brevity, and thoroughness, as shown in Figure 1.

**Figure 1.**
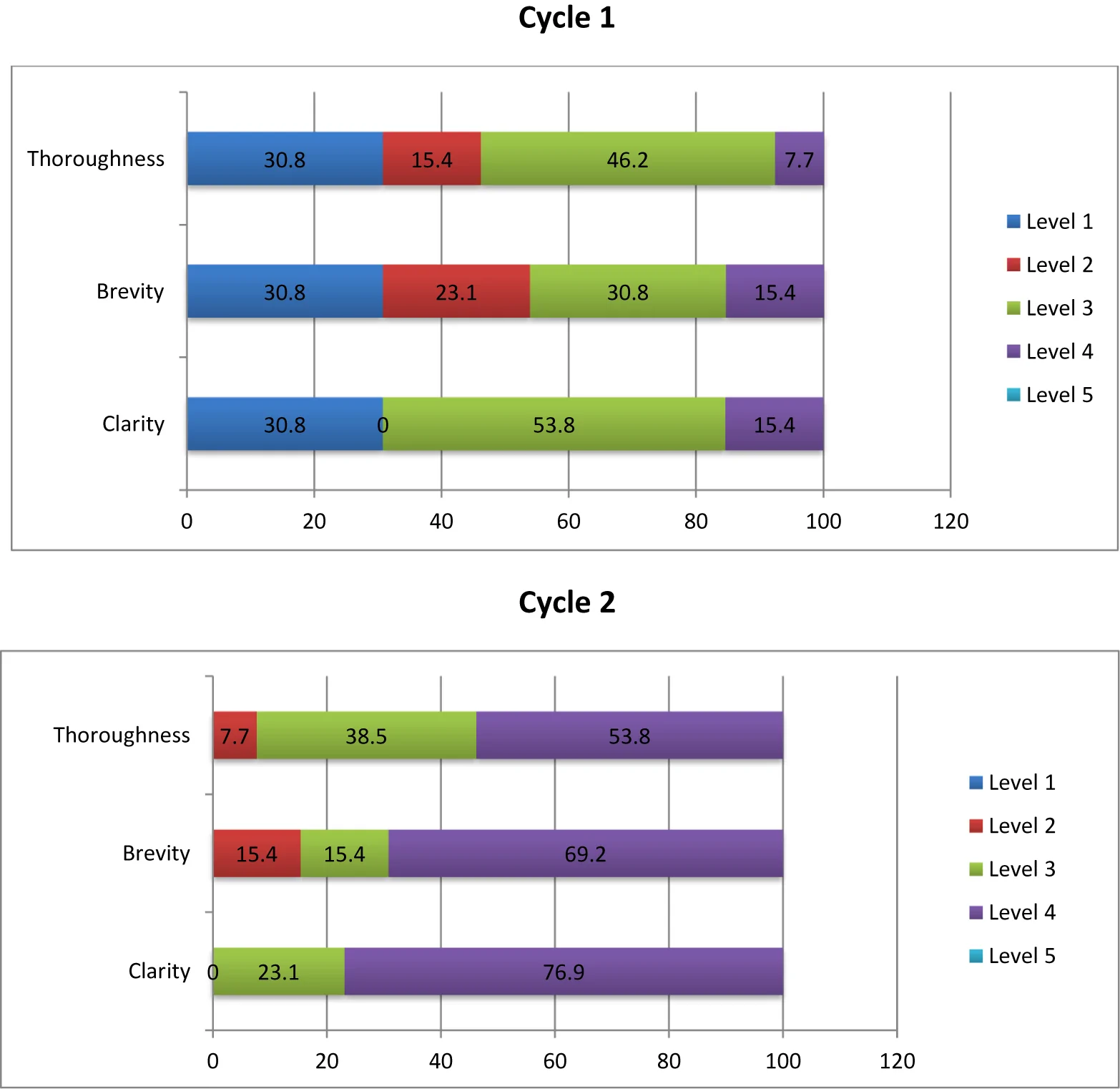
Perceptions of the quality of information transferred, rated on a 5-point Likert scale (1 = Poor, 5 = Excellent), showed a shift toward higher ratings over the cycle, indicating improved thoroughness, brevity, and clarity.

In the first cycle, the level of questioning during handovers was mostly rated at level 1 (53.8%) and level 2 (30.8%), with smaller proportions at level 3 (15.4%) and no ratings at levels 4 and 5. In the second cycle, ratings shifted, with the majority at level 2 (46.2%), followed by level 5 (23.1%), level 3 (15.4%), and level 4 (15.4%), and a small proportion remaining at level 1 (7.7%). Overall, there was an improvement in the level of questioning during handovers, with a shift from lower ratings in the first cycle to higher and more distributed ratings in the second cycle, as shown in Figure 2.

**Figure 2.**
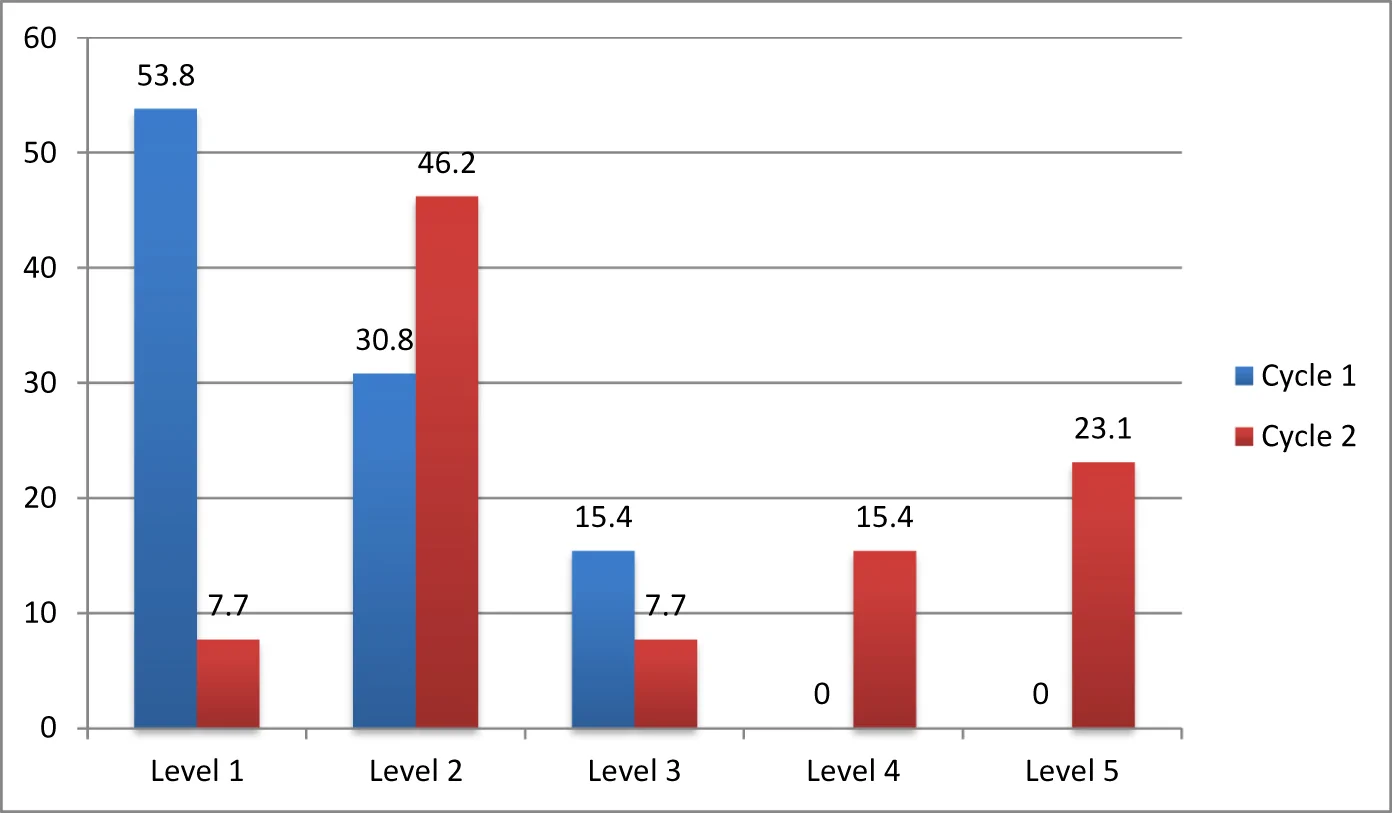
The level of questioning by junior doctors, assessed on a 5-point Likert scale (1 = poor, 5 = Excellent), indicated an increase in active participation and critical engagement during handovers in the second cycle.

## Discussion

This clinical audit compared handover practices in the ED and the Internal Medicine unit before and after implementing a standardized, I-PASS-based handover framework. The baseline audit revealed several flaws, including the lack of a standardized handover process, reliance on verbal communication, inconsistent communication of critical clinical information, and limited patient and family involvement. Following the implementation of the I-PASS framework, improvements were observed in adherence to standardized handover practices, communication of key clinical elements, synthesis by the receiving physician, patient and family involvement, and perceived communication quality.

The first audit cycle revealed a complete lack of standardized handover practice, with all participants (100%) reporting no structured format, all handovers being verbal (100%), and one-third (30.8%) being conducted via telephone. The lack of a standardized handover tool at baseline is consistent with previous research recommending structured handover protocols, such as I-PASS, to improve the consistency and completeness of information transfer while reducing communication failures^[^18,19^]^. The heavy reliance on verbal communication and telephone communication during handover can result in the loss of important information; this is consistent with previous studies describing handover as vulnerable to detail loss when it is based on memory or unstructured verbal exchange. Eggins and Slade observe that a lack of systematic structure and an overreliance on verbal communication contribute to incomplete handovers and communication risk^[^20^]^. About two-thirds of participants (69.2%) used bedside handover, which is widely promoted as a patient-centered strategy for improving accuracy and shared understanding. A systematic review of bedside handoff in intensive care found that it improved precision, reduced distractions, and decreased information loss, while a multicenter longitudinal study found that it was at least as safe as traditional handover^[^21^]^.

Baseline content elements, such as illness severity and patient summary, are frequently the easiest to communicate, so it’s no surprise that they were the most complete at baseline. In I-PASS, these are part of the “I” and “P” components, which are fundamental and frequently emphasized in structured handoffs^[^20^]^. The literature also shows that standardized handoff systems improve the inclusion of these fundamental elements when compared to unstructured verbal handoffs^[^22^]^. The moderate rates for differential diagnosis, chief complaint/timeframe, review of systems/physical findings, diagnostic results, medications administered, and consultations performed are consistent with evidence that handoff content varies greatly when no checklist is used. Studies of handoff quality note that important details are frequently omitted or inconsistently relayed, especially when the handoff relies on memory or informal practice^[^23^]^.

The post-intervention results show a significant improvement in handover practice following the implementation of the I-PASS structured tool. Complete adherence to I-PASS use, bedside handover, and standardized time compliance was achieved, along with 100% completion of all essential safety elements such as illness severity, patient summary, action plans, contingency planning, and receiver synthesis. These improvements are consistent with previous research demonstrating that structured handover tools improve information completeness, communication reliability, and patient safety^[^24,25^]^. The high level of patient/family involvement (92.3%) reflects the patient-centered nature of bedside handover approaches, which have been linked to increased engagement and transparency. The post-intervention results are especially important because they demonstrate that the handover process became not only more complete but also more participatory^[^26^]^. Full participation among physician roles suggests that the intervention was successfully implemented across the entire team^[^27^]^. Furthermore, the shift to higher quality ratings and increased questioning levels suggests improved communication quality and critical engagement during handover. Overall, these findings support the efficacy of combining structured communication tools with bedside practices to improve the consistency and quality of clinical handovers.

These findings indicate that a structured, low-cost handover intervention is feasible in this resource-constrained hospital and may serve as a good starting point for other institutions looking to standardize handover practice without investing heavily in technology or infrastructure. The intervention itself was low-cost and achievable within the existing infrastructure, relying solely on training sessions and a printed checklist rather than dedicated technology or protected educational time. However, due to the small, single-center sample, the short single-cycle follow-up interval, and the lack of clinical outcome data, these findings were analyzed descriptively rather than using formal paired statistical tests (e.g., McNemar’s or Wilcoxon signed-rank tests), and the results should be interpreted accordingly. Future audit cycles at this and similar institutions should include more follow-up than a single post-intervention assessment, multi-center designs, formal paired statistical testing where appropriate, and, where possible, the tracking of clinical outcome measures such as missed or delayed investigations, treatment delays, or adverse events to determine whether improved handover compliance translates into a measurable safety benefit.

## Limitations

This audit has several limitations that should be considered when interpreting the results. First, this audit was conducted at a single teaching hospital with a small sample size of participants, potentially limiting the statistical power and generalizability of the findings. The paired longitudinal design reduced inter-individual variability by assessing the same participants across both audit cycles; however, the results should be interpreted accordingly. Second, because the second cycle questionnaire was completed by the same thirteen participants, self-reported responses may be influenced by recall bias. Third, the audit was conducted in a limited timeframe, and the long-term viability of the observed improvements is unknown. Fourth, the audit assessed process compliance and perceived communication quality rather than clinical outcomes. As a result, while improved handover compliance may reduce the risk of communication-related harm, this study was unable to demonstrate a direct effect on clinical outcomes or patient safety and should be interpreted as a hypothesis for future outcome-focused research rather than a confirmed finding. Fifth, while the identical composition of respondents across both cycles creates a paired design suitable for formal paired statistical testing (e.g., McNemar’s test for binary outcomes, Wilcoxon signed-rank test for Likert-scale ratings), we chose to present findings descriptively due to the audit’s focus on quality improvement and small sample size. Future audits should include longer follow-up periods, structured reminder systems, set-aside time for questionnaire completion, and multi-center designs to validate and consolidate these findings.

## Conclusion

This audit discovered significant variation in baseline ED-to-medicine handover practices, such as inconsistent communication of safety-relevant information and limited patient involvement. Implementation of a structured I-PASS-based framework resulted in significant improvements in handover compliance, completeness, clarity, accountability, multidisciplinary engagement, and patient participation. Because this audit focused on process compliance and perceived communication quality rather than clinical outcomes such as adverse events, medication errors, missed investigations, or delayed treatment, the findings should be interpreted as evidence of improved handover compliance and perceived communication quality, rather than a demonstrated improvement in patient safety outcomes. Importantly, these gains were achieved through a simple, low-cost intervention in a resource-constrained environment. To confirm and extend these findings, long-term monitoring, institutional integration, periodic re-audits, and future studies incorporating clinical outcome measures are required.

## Data Availability

Data are provided within the manuscript.

## Research registration unique identifying number (UIN)

Al Qadarif Teaching Hospital Research Ethics Committee (Approval Number: 25/4/2024).
